# Diverse Heat Tolerance of the Yeast Symbionts of *Platycerus* Stag Beetles in Japan

**DOI:** 10.3389/fmicb.2021.793592

**Published:** 2022-01-07

**Authors:** Xue-Jiao Zhu, Sheng-Nan Zhang, Kana Watanabe, Kako Kawakami, Noriko Kubota, Etsuro Takagi, Masahiko Tanahashi, Xiu-Jun Wen, Kôhei Kubota

**Affiliations:** ^1^Laboratory of Forest Zoology, Department of Forest Science, Graduate School of Agricultural and Life Sciences, The University of Tokyo, Tokyo, Japan; ^2^Guandong Key Laboratory for Innovative Development and Utilization of Forest Plant Germplasm, College of Forestry and Landscape Architecture, South China Agricultural University, Guangzhou, China; ^3^Laboratory of Forest Zoology, Course of Applied Life Sciences, Faculty of Agriculture, The University of Tokyo, Tokyo, Japan; ^4^Independent Researcher, Kashiwa, Japan; ^5^Department of Tourism Science, Graduate School of Urban Environmental Sciences, Tokyo Metropolitan University, Hachioji, Japan; ^6^Department of Life Science, National Taiwan Normal University, Taipei, Taiwan; ^7^Bioproduction Research Institute, National Institute of Advanced Industrial Science and Technology, Tsukuba, Japan

**Keywords:** maximum growth temperature, maximum survival temperature, environmental factor, host wood material, *Scheffersomyces*

## Abstract

The genus *Platycerus* (Coleoptera: Lucanidae) is a small stag beetle group, which is adapted to cool-temperate deciduous broad-leaved forests in East Asia. Ten *Platycerus* species in Japan form a monophyletic clade endemic to Japan and inhabit species-specific climatic zones. They are reported to have co-evolutionary associations with their yeast symbionts of the genus *Sheffersomyces* based on host cytochrome oxidase subunit I (COI) and yeast intergenic spacer (IGS) phylogenies. Here we examined the heat tolerances of the yeast colonies isolated from the mycangia of 37 females belonging ten Japanese *Platycerus* species. The upper limits of growth and survival temperatures of each colony were decided by cultivating it at ten temperature levels between 17.5 and 40°C. Although both temperatures varied during 25.0–31.25°C, the maximum survival temperatures (MSTs) were a little higher than the maximum growth temperatures (MGTs) in 16 colonies. Pearson’s correlations between these temperatures and environmental factors (elevation and 19 bioclimatic variables from Worldclim database) of host beetle collection sites were calculated. These temperatures were significantly correlated with elevation negatively, the maximum temperature of the warmest month (Bio5) positively, and some precipitative variables, especially in the warm season (Bio12, 13, 16, 18) negatively. Sympatric *Platycerus kawadai* and *Platycerus albisomni* share the same lineage of yeast symbionts that exhibit the same heat tolerance, but the elevational lower range limit of *P. kawadai* is higher than that of *P. albisomni*. Based on the field survey in their sympatric site, the maximum temperature of host wood of *P. kawadai* larvae is higher about 2–3°C than that of *P. albisomni* larvae in the summer, which may restrict the elevational range of *P. kawadai* to higher area. In conclusion, it is suggested that the heat tolerance of yeast symbionts restricts the habitat range of their host *Platycerus* species or/and that the environmental condition that host *Platycerus* species prefers affect the heat tolerance of its yeast symbionts.

## Introduction

Diverse insect taxa and fungi can have mutualistic relationships (Biedermann and Vega, 2020). The presence of mycangia in insects, which are fungus-carrying organs, indicates obligate dependencies of the insects on fungal functions (Mueller et al., 1998; Aanen et al., 2009; Grebennikov and Leschen, 2010). Symbiotic relationships with fungi are especially critical for wood-feeding insects, because wood consists of polymers that are indigestible to insects such as cellulose, hemicellulose, and lignin (Haack and Slansky, 1987; Geib et al., 2008; Stokland, 2012). Female stag beetles (family Lucanidae) possess mycangia in the form of an exoskeletal organ on the dorsal side of the abdominal tip that carries microbial symbionts (Tanahashi et al., 2010; Tanahashi and Hawes, 2016; Kubota et al., 2020). Stag beetles mainly feed on decaying wood (Tanahashi and Kubota, 2013; Tanahashi et al., 2018) and commonly carry yeast symbionts belonging to the genus *Scheffersomyces*, a xylose-fermenting group of yeasts (Du Preez and Prior, 1985; Jeffries and Kurtzman, 1994; Olsson and Hahn-Hägerdal, 1996). Xylose is the main component of hemicellulose in broad-leaved tree species (Sjöström, 1993).

*Platycerus* is a genus of small stag beetles, and *Platycerus* species have adapted to mature cool-temperate, deciduous broad-leaved forests in East Asia (Kubota et al., 2011; Zhu et al., 2019, 2020). Ten *Platycerus* species in Japan form a monophyletic clade endemic to Japan and inhabit species-specific climatic zones (Kubota et al., 2011; Zhang and Kubota, 2021a,b). Their mycangia contain *Scheffersomyces* yeast symbionts that are closely related to *Scheffersomyces segobiensis*. Most known yeast symbionts of *Dorcus*, *Lucanus*, and *Prismognathus* stag beetle species besides *Platycerus* are also related to *S. segobiensis* or *Scheffersomyces stipitis* (Tanahashi et al., 2017; Kubota et al., 2020; Zhu et al., 2020; Ueki et al., 2021). Based on phylogenetic analyses of insect cytochrome oxidase subunit I (COI) and yeast intergenic spacer (IGS) genes, Japanese *Platycerus* have co-evolutionary associations with their yeast symbionts, which indicates an obligative dependency between *Platycerus* species and *Scheffersomyces* yeasts, although their contribution to host beetle growth and development has not been clarified (Tanahashi et al., 2017; Kubota et al., 2020). The adaptation of *Platycerus* species to cool temperate forests may be related to the heat tolerance of the beetles or microbial symbionts.

Climate change is one of the most serious issues of the present and near future, and it affects species distributions (Hamann and Wang, 2006; McKenney et al., 2007; Tonnang et al., 2010; Qin et al., 2019) and species interactions (Gilman et al., 2010; Walther, 2010; Harley, 2011; Blois et al., 2013). Its negative effects on mutualistic relationships with microbes have recently gained increasing attention (Wernegreen, 2012; Kikuchi et al., 2016; Hughes et al., 2017). *Platycerus* species in Japan are adapted to a cool climate and are considered somewhat endangered by global warming (Kubota et al., 2010). The Representative Concentration Pathway 8.5 (RCP 8.5) scenario predicts a major distribution range loss for *Platycerus* species by 2070 (Zhang and Kubota, 2021a). Intraspecific variation may also be important for local adaptation by the species (Zhang and Kubota, 2021b). However, these predictions do not reflect the temperature tolerance of microbial symbionts such as *Scheffersomyces* yeasts. We consider the yeast symbionts may affect the climate adaptation of their *Platycerus* host species.

To better understand climate adaptation of Japanese *Platycerus* species, it is expected to evaluate heat tolerance of their likely species-specific *Scheffersomyces* yeast symbionts. We estimated two temperature tolerance indicators for the yeast symbionts and examined the relationships between the heat tolerance and environmental factors at the beetle collection sites. We focused on three sympatric species and investigated the characteristics and seasonal temperature changes of the host wood materials. Finally, we discuss the relationship between Japanese *Platycerus* species and their yeast symbionts regarding climate adaptation.

## Materials and Methods

### Heat Tolerance Indicators

We examined the heat tolerance levels of 37 yeast strains from *Platycerus* species, of which 36 were obtained in a previous study by Kubota et al. (2020) and one was newly extracted from *Platycerus kawadai*. As a control group, we used 12 yeast strains from other lucanid genera obtained in previous studies (11 from Kubota et al., 2020 and one from Zhu et al., 2020) (Table 1, Supplementary Table 1, and Supplementary Figure 1).

**TABLE 1:** Samples used to determine the maximum growth temperature (MGT) and maximum survival temperature (MST).

Lucanid taxon	Site no.	Yeast strain	IGS Clade	Strain no. on the plate	MGT (°C)	MST (°C)
*Pltycerus acuticollis* K. Kurosawa, 1969	5	YW07.8	Clade Ia	1	28.75	28.75
	10	YW06.1	Clade Ia	2	28.75	31.25
*P. albisomni* Kubota, Kubota et Otobe, 2008	3	YW19.1	Clade Ia	3	28.75	28.75
	15	YW23.1	Clade Ia	4	28.75	28.75
ssp. *chichibuensis* Kubota, Kubota et Otobe, 2008	6	YW08.1	Clade Ia	5	28.75	30
	7	YW09.1	Clade Ia	6	28.75	30
*P. takakuwai* Fujita, 1987	11	YW57.1	Clade Ia	7	28.75	30
	13	YW86.3	Clade Ia	8	28.75	30
	14	YW13.1	Clade Ia	9	28.75	30
ssp. *akitai* Fujita, 1987	17	YW73.3	Clade Ia	10	27.5	27.5
	19	YW38.1	Clade Ia	11	28.75	30
	21	YW88.1	Clade Ib	12	28.75	28.75
ssp. *namedai* Fujita, 1987	25	YW43.2	Clade Ic, Shikoku	13	28.75	28.75
*P. viridicuprus* Kubota, Kubota et Otobe, 2008	22	YW76.3	Clade II	14	31.25	31.25
	23	YW15.1	Clade II	15	31.25	31.25
	24	YW78.3	Clade II	16	31.25	31.25
ssp. *kanadai* Kubota, Kubota et Otobe, 2008	30	YW58.1	Clade II	17	31.25	31.25
	32	YW87.1	Clade Ic, Kyushu	18	26.25	26.25
*P. akitaorum* Imura, 2007	20	YW14.9	Clade Ib	19	26.25	26.25
*P. sugitai* Okuda et Fujita, 1987	26	YW44.1	Clade Ic, Shikoku	20	26.25	26.25
	28	YW81.3	Clade Ic, Shikoku	21	26.25	27.5
*P. urushiyamai* Imura, 2007	31	YW48.1	Clade Ic, Kyushu	22	25	25
	36	YW50.1	Clade Ic, Kyushu	23	26.25	26.25
*P. sue* Imura, 2007	29	YW45.1	Clade Ic, Shikoku	24	26.25	26.25
*P. delicatulus* Lewis, 1883	5	YW65.2	Clade Id	25	28.75	31.25
	7	YW10.1	Clade Id	26	28.75	28.75
	11	YW52.1	Clade Id	27	28.75	28.75
	27	YW46.1	Clade Id	28	28.75	28.75
	32	YW72.2	Clade Id	29	28.75	30
	33	YW61.2	Clade Id	30	28.75	30
	34	YW60.2	Clade Id	31	28.75	30
ssp. *unzendakensis* Fujita et Ichikawa, 1982	35	YW47.2	Clade Id	32	28.75	30
*P. kawadai* Fujita et Ichikawa, 1982	7	YC055.9	Clade Ia	33	28.75	30
	11	YW53.1	Clade Ia	34	30	30
	13	YW03.1	Clade Ia	35	28.75	30
	14	YW12.1	Clade Ia	36	28.75	28.75
	16	YW70.3	Clade Ia	37	28.75	30
*Prismognathus angularis* Waterhouse, 1874	20	YW25.8	–	38	31.25	31.25
	12	YC021.5	–	39	31.25	31.25
*Lucanus maculifemoratus* Motsulsky, 1861	4	YW31.1	–	40	28.75	28.75
*Dorcus hopei binodulosus* Waterhouse, 1874	4	YW42.1	–	41	38.75	38.75
*D. rectus* (Motchulsky, 1857)	8	YW01.2	–	42	36.25	36.25
*D. titanus pilifer* Vollenhoven, 1861	9	YW63.1	–	43	36.25	36.25
*D. montivagus* (Lewis, 1883)	2	YW26.1	–	44	31.25	31.25
*D. rubrofemoratus* (Vollenhoven, 1865)	1	YW27.1	–	45	33.75	33.75
	4	YW41.1	–	46	33.75	33.75
*D. striatipennis* (Motchulsky, 1861)	2	YW29.2	–	47	33.75	33.75
	4	YW40.1	–	48	28.75	28.75
*Figulus binodulus* Waterhouse, 1873	18	YW71.1	–	49	40	>*40*

**FIGURE 1:**
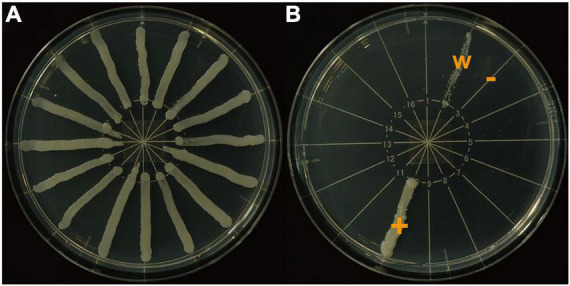
Yeast nitrogen base plates used to determine threshold temperatures. **(A)**, all colonies growing well (*n* = 16); **(B)**, colonies were divided into three growth categories: +, growing well; −, not growing; w, growing weakly.

Each frozen stock strain was thawed and spread onto a potato dextrose agar (Sigma Aldrich, St. Louis, MO, United States) plate. A small pellet of each colony was cultured in yeast and malt liquid medium (3.0 g/L yeast extract, 10.0 g/L malt extract, 10.0 g/L glucose) for 24 h followed by centrifugation at 2,000 rpm for 5 min and transfer of the precipitate into sterilized water. We adjusted the concentration of this suspension so that the optical density of each sample was 1.0 as measured using a spectrophotometer (V-630, JASCO Corporation, Hachioji, Japan) at a wavelength of 600 nm (OD600). We spread 1.0 μL of the prepared yeast suspension linearly onto a yeast nitrogen base (YNB) (1.6% YNB: Sigma Aldrich, St. Louis, MO, United States; 2% agar, 1.6% glucose) plate (Figure 1A). At most, 16 strains were put on one plate. The upper limit of growth and survival temperature of each strain were decided by cultivating each strain at ten temperatures between 17.5 and 40°C with 2.5°C intervals, since the temperature gradient incubator (TG-180-CCFL, NKsystems, Osaka) used in this study maintains accurate temperatures with setting 5°C intervals.

We used two heat tolerance indicators. We observed the growth condition of each yeast strain at four days after the beginning of incubation and classified the growth condition into the following three categories: +, growing well; −, not growing; w, growing weakly (Figure 1B). When + was followed by - over two consecutive temperature levels, we defined the mid-point temperature between the two levels as the maximum growth temperature (MGT) of that strain. When w was observed at a temperature level, we determined that temperature as the MGT for that strain.

Yeasts not growing at higher temperatures were not necessarily dead. Therefore, as the second indicator, we changed the incubating temperature of all yeasts to 20.0°C immediately after determining the MGT, which is a suitable growth temperature for all lucanid yeast symbionts used in this study. At two days after the temperature change, we observed the growth condition of each yeast strain again. We determined the maximum survival temperature (MST) using the same criteria as for the MGT (Figure 1B). The incubation days for elucidating both indicators were determined based on a preliminary experiment that identified the minimum incubation time needed for stable yeast colony formation.

### Correlations Between Heat Tolerance and Environmental Variables

We obtained environmental variables, including elevation and 19 bioclimatic variables covering the environmental niches for each studied stag beetle species, for each sampling site from Worldclim database^1^ (Hijmans et al., 2005) at a resolution of 2.5 arc-min (∼5 km) (Table 2). Then, we calculated Pearson correlation coefficients between environmental variables and the heat tolerance indicators of *Scheffersomyces* yeasts (MST and MGT) using R v. 3.6.3 (R Core Team, 2014).

**TABLE 2:** List of environmental variables examined in this study and Pearson correlation coefficients with the maximum growth temperature (MGT) and maximum survival temperature (MST).

Code	Environmental variables	Unit	Pearson correlation coefficients with heat tolerance of the yeast symbionts			
			MGT		MST	
Ele	Elevation	m	−0.33	*	−0.34	*
Bio1	Annual mean temperature	°C	0.13		0.11	
Bio2	Mean diurnal range [Mean of monthly (max temp–min temp)]	°C	0.29		0.41	*
Bio3	Isothermality (Bio2/Bio7) (* 100)	–	0.25		0.36	*
Bio4	Temperature Seasonality (standard deviation *100)	–	0.11		0.08	
Bio5	Max temperature of warmest month	°C	0.38	*	0.38	*
Bio6	Min temperature of coldest month	°C	0.07		0.02	
Bio7	Temperature annual range (Bio5-Bio6)	°C	0.26		0.33	*
Bio8	Mean temperature of wettest quarter	°C	0.31		0.22	
Bio9	Mean temperature of driest quarter	°C	0.15		0.09	
Bio10	Mean temperature of warmest quarter	°C	0.22		0.19	
Bio11	Mean temperature of coldest quarter	°C	0.11		0.1	
Bio12	Annual precipitation	mm	−0.49	**	−0.52	**
Bio13	Precipitation of wettest month	mm	−0.46	**	−0.42	***
Bio14	Precipitation of driest month	mm	0.12		−0.07	
Bio15	Precipitation seasonality (Coefficient of variation)	mm	−0.41	**	−0.22	
Bio16	Precipitation of wettest quarter	mm	−0.52	***	−0.48	***
Bio17	Precipitation of driest quarter	mm	0.05		−0.14	
Bio18	Precipitation of warmest quarter	mm	−0.54	***	−0.49	***
Bio19	Precipitation of coldest quarter	mm	0.09		−0.08	

*P < 0.05; **P < 0.01; ***P < 0.001.

### Characterization of Host Wood Materials of Sympatric *Platycerus* Species

On March 25, 30, and 31, and April 28, 2007, we examined the characteristics of host wood materials hosting the sympatric *Platycerus* species: *Platycerus delicatulus*, *Platycerus kawadai*, and *Platycerus albisomni* in the Irikawa area (Site 7 in Supplementary Table 1 and Supplementary Figure 1, about 150 m × 150 m and 1,300 m elevation) in the University of Tokyo Chichibu Forest, Saitama Prefecture, since it is one of locations where the maximum number (three) of *Platycerus* species can be found sympatrically in Japan (Zhang and Kubota, 2021a). Mature deciduous broad-leaved trees are dominant in the Irikawa area, such as *Fagus crenata*, *Fagus japonica*, *Quercus crispula*, *Prunus sargentii*, and *Acer* spp. The stag beetles *P. delicatulus* and *P. albisomni* are distributed above about 1,100 m elevation in the Irikawa area, whereas *P. kawadai* is restricted to elevations above 1,300 m.

We randomly chose 93 fallen or standing dead woods (branches and stems) of broad-leaved tree species and recorded their rot type, length, maximum diameter (except protruding branches), minimum height above the ground, maximum height above the ground, hard part ratio, and remaining bark ratio. The rot type was classified as white rot, brown rot, soft rot, or an intermediate type between different rot types. The hard part ratio represented the ratio of wood volume that could not be broken by hand. The hard part ratio and the remaining bark ratio were roughly estimated to the nearest 5% by eye. Following the random sampling, we searched for individuals of the three *Platycerus* species by looking for their oviposition marks on the wood surface and cutting wood by hatchet. When we found adults and larvae, we recorded their positions (depth below the wood surface, height above ground, and diameter of the branches or trunks) as well as other wood characteristics as described above. The collected larvae were reared and emerged adults were examined together with collected adults.

### Temperature Changes in Host Wood Materials of Sympatric *Platycerus* Species

We recorded the temperatures of five host wood materials in the Irikawa area that typically host the three sympatric *Platycerus* species, *P. delicatulus* (2), *P. kawadai* (1), and *P. albisomni* (2). We installed data loggers (Ondotori TR51i, T & D Corporation, Matsumoto, Japan) that record wood temperature every 30 min on the wood materials on May 12, 2015 and collected the data on November 12, 2015. This period includes the warmest season when *Platycerus* individuals are within their host wood at the egg or larval stage. To evaluate the relationship with the heat tolerance levels of yeast symbionts to increasing temperatures, we took the measurements during the warmest season. Each temperature sensor was installed at a depth of 1 cm at oviposition marks on the wood surface, where larvae are often found. The conditions of the surveyed wood materials are shown in Table 3 (also see Supplementary Figure 2).

**TABLE 3:** Host wood materials of three sympatric *Platycerus* species in which temperature changes were investigated in the Irikawa area, the University of Tokyo Chichibu Forest (1,300 m elevation).

Host species	No.	Height where the sensor was installed above the ground (cm)	Diameter of the host wood where the sensor was installed (cm)	Maximum temperature during surveyed period (°C)
*P. delicatulus*	1	155	23	24.6
*P. delicatulus*	2	90	17	24.3
*P. kawadai*		70	7	25.7
*P. albisomni*	1	0	11	23.9
*P. albisomni*	2	0	7	22.4

## Results

### Heat Tolerance Indicators

**FIGURE 2:**
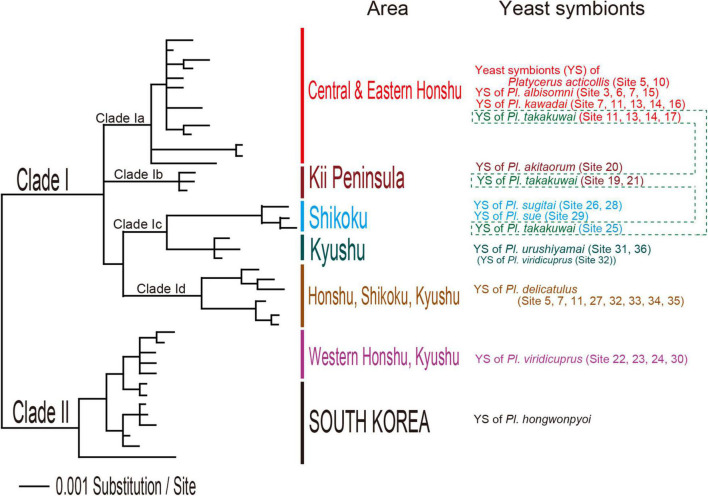
Bayesian inference (BI) phylogeny of yeast symbionts of *Platycerus* species in Japan based on intergenic spacer (IGS) sequences. The IGS sequences of South Korean *Platycerus hongwonpyoi* yeast symbionts were added as the outgroup (modified from Kubota et al., 2020).

The cultivation plate conditions at four days after the beginning of incubation, which was used to determine the MGT of *Scheffersomyces* yeasts, are shown in Supplementary Figures 3, 4. The plate conditions at two days after the incubation temperature change to 20.0°C, which was used to determine the MST, are shown in Supplementary Figures 5, 6. The yeast symbionts of all well examined lucanid species grew from 20.0 to 22.25°C. The MGT and MST of the yeast symbionts of each *Platycerus* species ranged between 25.0 and 31.25°C, and the MST was higher than the MGT in 16 strains. Symbionts of the *sugitai* species group (*Platycerus akitaorum*, *Platycerus sugitai*, and *Platycerus urushiyamai*) and *P*. *sue* (Clade Ic according to IGS based phylogeny; Table 1 and Figure 2) were relatively vulnerable to high temperatures (MGT: 25.0–27.25°C; MST: 25.0–27.5°C). By contrast, the symbionts of *Platycerus viridicuprus* (Clade II according to IGS based phylogeny; Table 1 and Figure 2) were resistant to high temperatures (MGT: 31.25°C; MST: 31.25°C) except for YS87.1 (Clade Ic according to IGS based phylogeny; Table 1 and Figure 2) (MGT: 26.25°C; MST: 26.25°C). Symbionts shared between the sympatric the *acuticollis* species group (*P. albisomni* and *Platycerus takakuwai*) and *P. kawadai* exhibited almost equally heat tolerance levels (MGT: 28.75–30.0°C; MST: 28.75–30.0°C). Their heat tolerance levels were almost the same but sometimes different from those of sympatric *P. delicatulus* (Clade Id according to IGS based phylogeny; Table 1 and Figure 2) (MGT: 28.75°C; MST: 28.75–31.25°C). Most yeast symbionts of other lucanid genera exhibited even higher temperature tolerance levels (MGT: 28.75–40.0°C; MST: 28.75 to >40.0°C) than those of *Platycerus* (Table 1).

### Correlations Between Heat Tolerance and Environmental Variables

Pearson correlation coefficients between environmental variables and the heat tolerance indicators of yeasts (MST and MGT) are shown in Table 1. Significant correlations are also shown in Supplementary Figures 7, 8. Strong negative correlations were observed between the indicators and precipitation-related variables (Bio12, Bio13, Bio16, and Bio18), especially in the warm season (Table 2). Temperature-related variables (Bio2, Bio3, Bio5, and Bio7) were positively related to MST. However, only one temperature related variables (Bio5; maximum temperature in the warmest month) was related to MGT. Moreover, negative correlations were observed between elevation and MGT and MST (Table 2).

### Characterization of the Host Wood Materials of Sympatric *Platycerus* Species

We found 31 adults (*P. delicatulus*: 18; *P. kawadai*: 9; *P. albisomni*: 4) and 79 larvae of *Platycerus* were found in 40 dead host wood materials. Of 79 larvae, 21 died and 58 emerged as adults (*P. delicatulus*: 32; *P. kawadai*: 16; *P. albisomni*: 21) in the laboratory from August to October of 2007. We identified the adult species based on their external morphologies, but could not identify the species of the dead larvae. Ultimately, we identified *Platycerus* species from 29 dead host wood materials. The numbers of wood materials that hosted *P. delicatulus*, *P. kawadai*, and *P. albisomni* were 13, 7, and 11, respectively (Supplementary Table 2). Both *P. delicatulus* and *P. kawadai* inhabited in two wood materials. Four tree species were identified as the host of *Platycerus* species (*F. japonica*, *F. crenata*, *P. sargentii*, and *Acer* sp.). However, most of the host tree species could not be identified. *Platycerus* females usually leave oviposition marks on the host wood. We found oviposition marks on 34 of 40 wood materials in which *Platycerus* adults or larvae were found.

The characteristics of the host wood of three species and randomly chosen wood materials are shown in Supplementary Table 2. Compared to randomly sampled wood materials, *P. delicatulus* preferred thicker wood, which is found at high positions above the ground. *P. kawadai* avoided wood on or under the ground and also preferred wood at high positions. *P. albisomni* preferred soft wood, which is found more commonly on or under the ground.

Of the host wood characteristics, the maximum diameter, hard part ratio, and maximum height above the ground were significantly different among species (Supplementary Table 2). Characteristic host wood values were similar between *P. delicatulus* and *P. kawadai*. The host wood of *P. albisomni* was much softer than that of *P. delicatulus* and *P. kawadai*. The maximum diameter and maximum height of the host wood of *P. albisomni* were significantly smaller and lower than those of *P. delicatulus*, respectively. Whereas the rot type of the host wood of *P. delicatulus* and *P. kawadai* was white rot or white-brown rot, that of the host wood of *P. albisomni* was white rot, soft rot, and white-soft rot. *Platycerus* species were not found in wood with brown rot or brown-soft rot (Supplementary Table 2). *P. albisomni* individuals lived in low-positioned host wood nearer to the ground, where the host wood was considerably wetter than that of *P. delicatulus* and *P. kawadai* (Supplementary Table 3).

### Temperature Changes in the Host Wood Materials of Sympatric *Platycerus* Species

The temperatures of five host wood materials were recorded smoothly from May 5 to August 31, 2021, whereas the temperature measurement occasionally failed after this period. The maximum temperatures of the five host wood materials varied between 12.0 and 25.7°C. The variation in diurnal maximum temperatures among host wood materials tended to be larger around the troughs and peaks (Supplementary Figure 9A). Because the highest temperature of each host wood was recorded on July 26 and 27 and August 1 and 2, we focused on temperature changes in each host wood from July 24 to August 2 (Supplementary Figure 9B). The maximum temperature of the host wood of *P. kawadai* larvae was about 2–3°C higher than that of *P. albisomni* larvae, and that of *P. delicatulus* was intermediate between the two species (Supplementary Figure 9B). Additionally, multiple sudden temperature changes were recorded, possibly caused by rain showers or changes in sunlight (Supplementary Figure 9B).

## Discussion

In this study, we demonstrated the variation in heat tolerance of the *Scheffersomyces* yeast symbionts of ten Japanese *Platycerus* species (Table 1). The heat tolerance levels (MGT and MST) seemed to be related to each yeast’s phylogenetic position based on the IGS gene, although statistical confirmation was limited because of the small sample size per clade except for Clade Ia (Table 1). Both MGT and MST were negatively correlated with elevation and positively correlated with the maximum temperature of the warmest month (Bio5). They were also negatively correlated with multiple precipitation-related variables, especially in the warm season (Bio12, 13, 16, and 18). Rainfall in the warm season lowers the atmosphere and habitat temperature, which may contribute to the survival of species without high heat tolerance. Over all, the habitat climate of *Platycerus* species is generally concordant with the heat tolerance of its yeast symbionts.

In eastern Japan, *P. delicatulus*, *P. kawadai*, and *P. albisomni* are sympatric and they prefer different types of host wood; *P. delicatulus* prefers relatively thick and hard, often standing wood with white rot, *P. kawadai* prefers relatively thin and hard, often standing wood with white rot, and *P. albisomni* prefers relatively thin and soft wood with white rot or soft rot on the ground (Imura, 2010). Little quantitative information about the host wood characteristics of *Platycerus* species was previously known (Ikeda, 1987). In this study, we quantitatively evaluated wood characteristics in the field (Supplementary Tables 2, 3), which are concordant with the available information (Imura, 2010). We analyzed the host wood materials of three species reflecting these characteristics (Table 3) and measured temperature changes in wood with oviposition marks. We focused on the maximum temperatures of host wood in the warm season to assess the heat tolerance of the yeast symbionts. The temperature fluctuations in the host wood of *P. kawadai* (thin and standing) and *P. albisomni* (on the ground) were the largest and smallest, respectively (Supplementary Figures 9A,B). The host wood of *P. albisomni* was typically wet, which might have contributed to its small temperature variation. As a result, the highest maximum host wood temperature during the study period was observed for *P. kawadai* (25.7°C), the lowest for *P. albisomni* (22.4 and 23.9°C), and an intermediate temperature for *P. delicatulus* (24.3 and 24.6°C) (Supplementary Figure 9B). The study site was near the lower elevational limits of *P. kawadai*, and the species’ maximum host wood temperature (25.7°C) was lower than its MGT and MST (28.75°C). Conclusively, the heat tolerance levels of the yeast symbionts are in accordance with the distribution of *P. delicatulus* and *P. albisomni* at lower elevations.

*Platycerus kawadai* and *P. albisomni* share the same lineage of yeast symbionts (Clade Ia according to IGS-based phylogeny), members of whom exhibit the same heat tolerance, whereas *P. delicatulus* hosts yeasts belonging to a different clade (Clade Id) (Figure 1). The difference between *P. kawadai* and *P. albisomni* in the maximum temperature of host wood reflects their yeast symbionts’ heat tolerance, which might explain their vertical distribution (*P. kawadai*: above 1,300 m elevation; *P. albisomni*: above 1,100 m elevation). If the two species exhibit the same heat tolerance as in their yeast symbionts at the larval stage in summer, the elevational lower limit of *P. kawadai* should be higher than that of *P. albisomni*, since the maximum temperature of the host wood of *P. kawadai* is higher than that of *P. albisomni* in the same site (Table 3). In conclusion, the heat tolerance of *Platycerus* yeast symbionts is concordant with climate conditions not only on the geographical level but also on the microhabitat level.

Our results suggest the following hypotheses regrading heat adaptation in *Platycerus* species: the heat tolerance of yeast symbionts restricts the habitat range of their host *Platycerus* species or/and the environmental condition that host *Platycerus* species prefers affect the heat tolerance of its yeast symbionts. For these hypotheses, it seems to be important to examine the heat tolerance of host beetles, or the temperature effect on yeast functions. However, the phylogeny of *Platycerus* species is not completely concordant with the phylogeny of their yeast symbionts (Kubota et al., 2020). This might be caused by the rare lateral transmission of yeasts (Ueki et al., 2021). Lateral transmission of yeast symbionts might have led to the evolution of thermal adaptation in *Platycerus* species, or the dispersal of *Platycerus* species to a new thermal environment might have result in lateral yeast transmission.

The family Lucanidae comprises more than 1,000 species, of which most species are distributed in warmer areas than *Platycerus* species (Kim and Farrell, 2015). In this study, most yeast symbionts of other lucanid taxa, including cool-adapted species (e.g., *Prismognathus angularis*) exhibited higher heat tolerance than did those of *Platycerus* species. Because they are distantly related to symbionts of *Platycerus* species (Kubota et al., 2020), this yeast-related phylogenetic constraint of stag beetles might have affected heat adaptation in *Scheffersomyces* yeasts. For the heat-tolerant yeasts such as *Saccharomyces cerevisiae* and *Kluyveromyces marxianus*, the molecular and metabolic bases of the response to heat stress have been examined (Piper, 1993; Kalyuzhin, 2011; Huang et al., 2018; Matsumoto et al., 2018). The factors exhibiting diverse heat tolerance in *Scheffersomyces* yeasts seem to be a future research topic.

Under climate change, the thermal sensitivity of microbial symbionts constrains insect responses, and highly dependent microbial mutualisms may strongly restrict thermal responses (Wernegreen, 2012) such as in southern green stinkbugs and bacterial gut symbionts (Kikuchi et al., 2016), or corals and zooxanthellae (Hughes et al., 2017). Therefore, rapid global warming is likely to promote a range shift, and local extinction of *Platycerus* species due to the heat tolerance of their yeast symbionts. It may also promote the replacement of yeast symbionts that exhibit low heat tolerance by more tolerant symbionts of *Platycerus* species or other lucanid taxa.

Surprisingly, *Scheffersomyces* is almost the only fungus that *Platycerus* and many lucanid taxa carry in their mycangia (Tanahashi et al., 2010, 2017; Kubota et al., 2020), which suggest the importance of *Scheffersomyces* to lucanid taxa. However, lucanid species are expected to additionally carry the diverse bacterial taxa, which may also affect the thermal response of the host beetles.

In conclusion, we suggested that there is a close relationship between thermal adaptation in *Scheffersomyces* yeasts and the environmental factors preferred by their *Platycerus* host species. This study’s results will contribute to a better understanding of the evolution of symbiotic lucanid-microbial systems.

## Data Availability Statement

The original contributions presented in the study are included in the article/Supplementary Material, further inquiries can be directed to the corresponding author.

## Author Contributions

X-JZ, KW, KKa, NK, ET, MT, and KKu contributed to the data generation of this study. S-NZ performed the environmental analysis. KKu contributed to the study design with the help of MT. X-JZ and KKu wrote the manuscript with help of X-JW. All authors approved the final version of the manuscript.

## Conflict of Interest

The authors declare that the research was conducted in the absence of any commercial or financial relationships that could be construed as a potential conflict of interest.

## Publisher’s Note

All claims expressed in this article are solely those of the authors and do not necessarily represent those of their affiliated organizations, or those of the publisher, the editors and the reviewers. Any product that may be evaluated in this article, or claim that may be made by its manufacturer, is not guaranteed or endorsed by the publisher.

## Footnotes

1
https://www.worldclim.org/

## References

[B1] Aanen D. K. de Fine Licht H. H. Debets A. J. Kerstes N. A. Hoekstra R. F. Boomsma J. J. (2009). High symbiont relatedness stabilizes mutualistic cooperation in fungus-growing termites. *Science* 326 1103–1106. 10.1126/science.1173462 19965427

[B2] Biedermann P. H. W. Vega F. E. (2020). Ecology and evolution of insect-fungus mutualisms. *Annu. Rev. Entomol*. 65 431–455. 10.1146/annurev-ento-011019-024910 31610133

[B3] Blois J. L. Zarnetske P. L. Fitzpatrick M. C. Finnegan S. (2013). Climate change and the past, present, and future of biotic interactions. *Science* 341 499–504. 10.1126/science.1237184 23908227

[B4] Du Preez J. C. Prior B. A. (1985). A quantitative screening of some xylose-fermenting yeast isolates. *Biotechnol. Lett.* 7 241–246.

[B5] Geib S. M. Filley T. R. Hatcher P. G. Hoover K. Carlson J. E. del Mar Jimenez-Gasco M. (2008). Lignin degradation in wood-feeding insects. *Proc. Natl. Acad. Sci. U.S.A.* 105 12932–12937. 10.1073/pnas.0805257105 18725643 PMC2529026

[B6] Gilman S. E. Urban M. C. Tewksbury J. Gilchrist G. W. Holt R. D. (2010). A framework for community interactions under climate change. *Trends Ecol. Evol.* 25 325–331. 10.1016/j.tree.2010.03.002 20392517

[B7] Grebennikov V. V. Leschen R. A. B. (2010). External exoskeletal cavities in Coleoptera and their possible mycangial functions. *Entomol*. *Sci*. 13 81–98. 10.1111/j.1479-8298.2009.00351.x

[B8] Haack R. A. Slansky F. (1987). “Nutritional ecology of wood-feeding Coleoptera, Lepidoptera, and Hymenoptera,” in *Nutritional Ecology of Insects, Mites, and Spiders*, eds Slansky F. Rodriguez J. G. (New York NY: John Wiley), 449–486.

[B9] Hamann A. Wang T. (2006). Potential effects of climate change on ecosystem and tree species distribution in British Columbia. *Ecology* 87 2773–2786. 17168022 10.1890/0012-9658(2006)87[2773:peocco]2.0.co;2

[B10] Harley C. D. (2011). Climate change, keystone predation, and biodiversity loss. *Science* 334 1124–1127. 10.1126/science.1210199 22116885

[B11] Hijmans R. J. Cameron S. E. Parra J. L. Jones P. G. Jarvis A. (2005). Very high resolution interpolated climate surfaces for global land areas. *Int. J. Climatol.* 25 1965–1978. 10.1002/joc.1276

[B12] Huang C.-J. Lu M.-Y. Chang Y.-W. Li W.-H. (2018). Experimental evolution of yeast for high-temperature tolerance. *Mol. Biol. Evol.* 35 1823–1839. 10.1093/molbev/msy077 29684163

[B13] Hughes T. P. Kerry J. T. Álvarez-Noriega M. Álvarez-Romero J. G. Anderson K. D. Baird A. H. (2017). Global warming and recurrent mass bleaching of corals. *Nature* 543 373–377. 10.1038/nature21707 28300113

[B14] Ikeda K. (1987). “Habitat segregation and distribution of the genus *Platycerus* in Japan,” in *Insect Communities in Japan*, eds Kimoto S. Takeda H. (Hiratsuka: Tokai University Press), 93–101.

[B15] Imura (2010). *The Genus Platycerus of East Asia.* Tokyo: Roppon-Ashi Entomological Books, (in Japanese, partly in English and Chinese).

[B16] Jeffries T. W. Kurtzman C. B. (1994). Strain selection, taxonomy, and genetics of 220 xylose-fermenting yeasts. *Enzyme Microb. Technol.* 16 922–932.

[B17] Kalyuzhin V. A. (2011). Heat resistance in *Saccharomyces cerevisiae* yeast. *Biol. Bull. Rev.* 1 207–213. 10.1134/S2079086411030042

[B18] Kikuchi Y. Tada A. Musolin D. L. Hari N. Hosokawa T. Fujisaki K. (2016). Collapse of insect gut symbiosis under simulated climate change. *mBio* 7:e01578-16. 10.1128/mBio.01578-16 27703075 PMC5050343

[B19] Kim S. I. Farrell B. D. (2015). Phylogeny of world stag beetles (Coleoptera: Lucanidae) reveals a Gondwanan origin of Darwin’s stag beetle. *Mol. Phylogenet. Evol*. 86 35–48. 10.1016/j.ympev.2015.02.015 25732069

[B20] Kubota K. Kubota N. Otobe H. (2010). Mitochondrial gene diversity of *Platycerus sue* (Coleoptera, Lucanidae), a temporarily designated endangered species based on the law for the conservation of endangered species of wild fauna and flora. *Bull. Biogeogr. Soc. Japan* 65 151–158 (in Japanese with English Abstract).

[B21] Kubota K. Nagahata Y. Ikeda H. Kubota N. Otobe H. Umetsu K. (2011). Diversification process of stag beetles belonging to the genus *Platycerus* Geoffroy (Coleoptera: Lucanidae) in Japan based on nuclear and mitochondrial genes. *Entomol. Sci.* 14 411–427. 10.1111/j.1479-8298.2011.00466.x

[B22] Kubota K. Watanabe K. Zhu X. J. Kawakami K. Tanahashi M. Fukatsu T. (2020). Evolutionary relationship between *Platycerus* stag beetles and their mycangium-associated yeast symbionts. *Front. Microbiol.* 11:1436. 10.3389/fmicb.2020.01436 32695086 PMC7338584

[B23] Matsumoto I. Arai T. Nishimoto Y. Leelavatcharamas V. Furuta M. Kishida M. (2018). Thermotolerant yeast *Kluyveromyces marxianus* reveals more tolerance to heat shock than the brewery yeast *Saccharomyces cerevisiae*. *Biocontrol Sci.* 23 133–138. 10.4265/bio.23.133 30249963

[B24] McKenney D. W. Pedlar J. H. Lawrence K. Campbell K. Hutchinson M. F. (2007). Potential impacts of climate change on the distribution of North American trees. *Bioscience* 57 939–948. 10.1641/B571106

[B25] Mueller U. G. Rehner S. A. Schultz T. R. (1998). The evolution of agriculture in ants. *Science* 281 2034–2038. 10.1126/science.281.5385.2034 9748164

[B26] Olsson L. Hahn-Hägerdal B. (1996). Fermentation of lignocellulosic hydrolysates for ethanol production. *Enzyme Microb. Technol.* 18 312–331. 10.1016/0141-0229(95)00157-3

[B27] Piper P. W. (1993). Molecular events associated with acquisition of heat tolerance by the yeast *Saccharomyces cerevisiae*. *FEMS Microbiol. Rev.* 11 339–355. 10.1111/j.1574-6976.1993.tb00005.x 8398211

[B28] Qin Y. Wang C. Zhao Z. Pan X. Li Z. (2019). Climate change impacts on the global potential geographical distribution of the agricultural invasive pest, *Bactrocera dorsalis* (Hendel) (Diptera: Tephritidae). *Clim. Change* 155 145–156. 10.1007/s10584-019-02460-3

[B29] R Core Team (2014). *R: A Language and Environment for Statistical Computing.* Vienna: R Foundation for Statistical Computing.

[B30] Sjöström E. (1993). *Wood Chemistry: Fundamentals and Applications*, 2nd Edn. San Diego CA: Academic Press.

[B31] Stokland J. N. (2012). “Wood decomposition,” in *Biodiversity in Decaying Wood*, eds Stokland J. N. Siitonen J. Jonsson B. G. (New York, NY: Cambridge University Press), 10–28. 10.1017/CBO9781139025843

[B32] Tanahashi M. Hawes C. J. (2016). The presence of a mycangium in the horned stag beetle *Sinodendron cylindricum* (Coleoptera: Lucanidae) and the associate yeast symbionts. *J. Insect Sci.* 16:76. 10.1093/jisesa/iew054 27432353 PMC4948600

[B33] Tanahashi M. Kubota K. (2013). Utilization of the nutrients in the soluble and insoluble fractions of fungal mycelium by larvae of the stag beetle, *Dorcus rectus* (Coleoptera: Lucanidae). *Eur. J. Entomol.* 110 611–615. 10.14411/eje.2013.083

[B34] Tanahashi M. Ikeda H. Kubota K. (2018). Elementary budget of stag beetle larvae associated with selective utilization of nitrogen in decaying wood. *Sci. Nat.* 105:33. 10.1007/s00114-018-1557-x 29725830

[B35] Tanahashi M. Kim J.-K. Watanabe K. Fukatsu T. Kubota K. (2017). Specificity and genetic diversity of xylose-fermenting *Scheffersomyces* yeasts associated with small blue stag beetles of the genus *Platycerus* in East Asia. *Mycologia* 109 630–642. 10.1080/00275514.1382648 29140770

[B36] Tanahashi M. Kubota K. Matsushita N. Togashi K. (2010). Discovery of mycangia and associated xylose-fermenting yeasts in stag beetles (Coleoptera: Lucanidae). *Naturwissenschaften* 97 311–317. 10.1007/s00114-009-0643-5 20107974

[B37] Tonnang H. E. Kangalawe R. Y. Yanda P. Z. (2010). Predicting and mapping malaria under climate change scenarios: the potential redistribution of malaria vectors in Africa. *Malar. J.* 9:111. 10.1186/1475-2875-9-111 20416059 PMC2873524

[B38] Ueki G. Zhang S.-N. Zhu X.-J. Wen X.-J. Tojo K. Kubota K. (2021). Lateral transmission of the yeast symbionts among lucanid beetle taxa. *Front. Microbiol.* 11:794904. 10.3389/fmicb.2021.794904 PMC8712881 34970248

[B39] Walther G. R. (2010). Community and ecosystem responses to recent climate change. *Philos. Trans. R. Soc. Lond. B Biol. Sci*. 365 2019–2024. 10.1098/rstb.2010.0021 20513710 PMC2880129

[B40] Wernegreen J. J. (2012). Mutualism meltdown in insects: bacteria constrain thermal adaptation. *Curr. Opin. Microbiol.* 15 255–262. 10.1016/j.mib.2012.02.001 22381679 PMC3590105

[B41] Zhang S.-N. Kubota K. (2021a). Dispersal constraints on the potential distribution of cold-adapted stag beetles (genus *Platycerus*) in Japan and the implications of climate change. *Insect Conserv. Divers.* 14 356–366. 10.1111/icad.12461

[B42] Zhang S.-N. Kubota K. (2021b). Integrating intraspecific variation in species distribution models by quantifying niche differentiation. *Biol. J. Linn. Soc.* 133 187–201. 10.1093/biolinnean/blab021

[B43] Zhu X. J. Jang T. W. Kim J. K. Kubota K. (2019). Genetic divergence of *Platycerus hongwonpyoi* (Coleoptera: Lucanidae) in South Korea. *Entomol. Sci.* 22 86–97. 10.1111/ens.12337

[B44] Zhu X.-J. Ma T. Imura Y. Wen X.-J. Kubota K. (2020). Molecular phylogeny and historical biogeography of the genus *Platycerus* (Coleoptera, Lucanidae) in East Asia. *Zool. Scr.* 49 582–595. 10.1111/zsc.12429

