# Supplementary material for: Diverse Heat Tolerance of the Yeast Symbionts of Platycerus Stag Beetles in Japan
Source: Front Microbiol. 2022 Jan 7;12:793592. doi: 10.3389/fmicb.2021.793592 (PMC8776712; doi:10.3389/fmicb.2021.793592)
Supplement: Supplementary file 1 [file Data_Sheet_1.PDF]

**Supplementary Table 1.** Sample collection sites.

| Site no. | Elevation | Latitude | Longitude |
|---|---|---|---|
| 1 | 80 | 41.89 | 140.33 |
| 2 | 200 | 41.72 | 140.29 |
| 3 | 640 | 38.48 | 140.01 |
| 4 | 780 | 37.06 | 139.52 |
| 5 | 1280 | 36.75 | 139.44 |
| 6 | 1100 | 36.15 | 138.90 |
| 7 | 1300 | 35.94 | 138.80 |
| 8 | 20 | 35.90 | 139.94 |
| 9 | 60 | 35.74 | 139.54 |
| 10 | 460 | 35.54 | 139.09 |
| 11 | 1400 | 35.51 | 139.05 |
| 13 | 1420 | 35.23 | 139.02 |
| 14 | 1200 | 34.85 | 138.96 |
| 15 | 1330 | 36.91 | 138.48 |
| 16 | 1640 | 35.55 | 138.09 |
| 17 | 1470 | 36.02 | 137.24 |
| 18 | 5 | 34.49 | 136.69 |
| 19 | 770 | 34.53 | 136.22 |
| 20 | 1520 | 34.19 | 136.10 |
| 21 | 1100 | 34.23 | 136.02 |
| 22 | 1000 | 35.44 | 134.46 |
| 23 | 450 | 36.26 | 133.33 |
| 24 | 1180 | 35.05 | 133.07 |
| 25 | 1030 | 33.92 | 134.29 |
| 26 | 1370 | 33.87 | 134.09 |
| 27 | 1320 | 33.87 | 134.09 |
| 28 | 1430 | 33.75 | 133.15 |
| 29 | 1680 | 33.77 | 133.12 |
| 30 | 680 | 33.75 | 130.80 |
| 31 | 960 | 33.48 | 130.93 |
| 32 | 740 | 33.46 | 130.91 |
| 33 | 1100 | 33.28 | 131.40 |
| 34 | 880 | 33.12 | 131.29 |
| 35 | 900 | 32.96 | 130.08 |
| 36 | 1370 | 32.16 | 130.94 |
