# Supplementary material for: Diverse Heat Tolerance of the Yeast Symbionts of Platycerus Stag Beetles in Japan
Source: Front Microbiol. 2022 Jan 7;12:793592. doi: 10.3389/fmicb.2021.793592 (PMC8776712; doi:10.3389/fmicb.2021.793592)
Supplement: Supplementary file 2 [file Data_Sheet_2.PDF]

Supplementary Table 2. Characteristics of host wood materials in which each beetle species was found and of randomly sampled dead, decaying wood in the Irikawa area, the University of Tokyo Chichibu Forest (1,300 m elevation).

Classification of twigs and trunks based on species found there (number examined)	Length (mean±SD cm)	Maximum diameter† (mean±SD cm)	Ratio of the hard part†† (mean±SD %)	Ratio of the remaining bark (mean±SD %)	Minimum height above the ground (mean±SD cm)	Maximum height above the ground † (mean±SD cm)	Rot type
Platycerus delicatulus (n = 13)	152.9±97.0	14.0±6.0*a	51.9±312.0a	17.7±20.9	11.6±39.2	92.5±83.0**a	white (11), white–brown (2)
P. kawadai (n = 7)	112.9±59.4	11.6±6.5ab	64.3±17.0a	37.9±26.3	26.3±43.1*	90.3+81.9*ab	white (4), white–brown (3)
P. albisomni chichibuensis (n = 11)	115.9±119.8	8.1±4.6b	19.1±31.5*b	12.3±26.2	–2.3±3.4**	7.5±5.6b	white (1), white–soft (7), soft (3)
Random sampling (n = 93)	93.1±100.7	8.9±6.9	40.5±35.9	27.8±33.7	2.3±18.8	20.7±44.2	white (39), brown (10), soft (15), white–brown (22), white–soft (4), brown–soft (3)

*, **, significant differences from random sampling; *, p<0.05; **, p<0.01; †, ††, significant differences among species; †, p<0.05; ††, p<0.01; different letters for each species indicate significant differences between species.
