# Supplementary material for: Diverse Heat Tolerance of the Yeast Symbionts of Platycerus Stag Beetles in Japan
Source: Front Microbiol. 2022 Jan 7;12:793592. doi: 10.3389/fmicb.2021.793592 (PMC8776712; doi:10.3389/fmicb.2021.793592)
Supplement: Supplementary file 3 [file Data_Sheet_3.PDF]

Supplementary Table 3. Characteristics of the positions in host wood materials in which individuals of each beetle species was found in the Irikawa area, the University of Tokyo Chichibu Forest (1,300 m elevation).

Species	Depth below the surface of woody materials** (mean±SD cm)	Height above the ground** (mean±SD cm)	Diameter of the woody materials (mean±SD cm)
Platycerus delicatulus (n = 50)	2.9±2.0a	38.7±28.5a	11.7±6.1
P. kawadai (n = 25)	2.5±1.5a	48.6±51.3a	9.3±4.6
P. albisomni chichibuensis (n = 25)	1.2±0.6b	0.0±8.0b	10.3±4.5

**, $p<0.01$; different letters indicate significant differences among species.
