# Supplementary material for: Diverse Heat Tolerance of the Yeast Symbionts of Platycerus Stag Beetles in Japan
Source: Front Microbiol. 2022 Jan 7;12:793592. doi: 10.3389/fmicb.2021.793592 (PMC8776712; doi:10.3389/fmicb.2021.793592)
Supplement: Supplementary file 4 [file Data_Sheet_4.PDF]

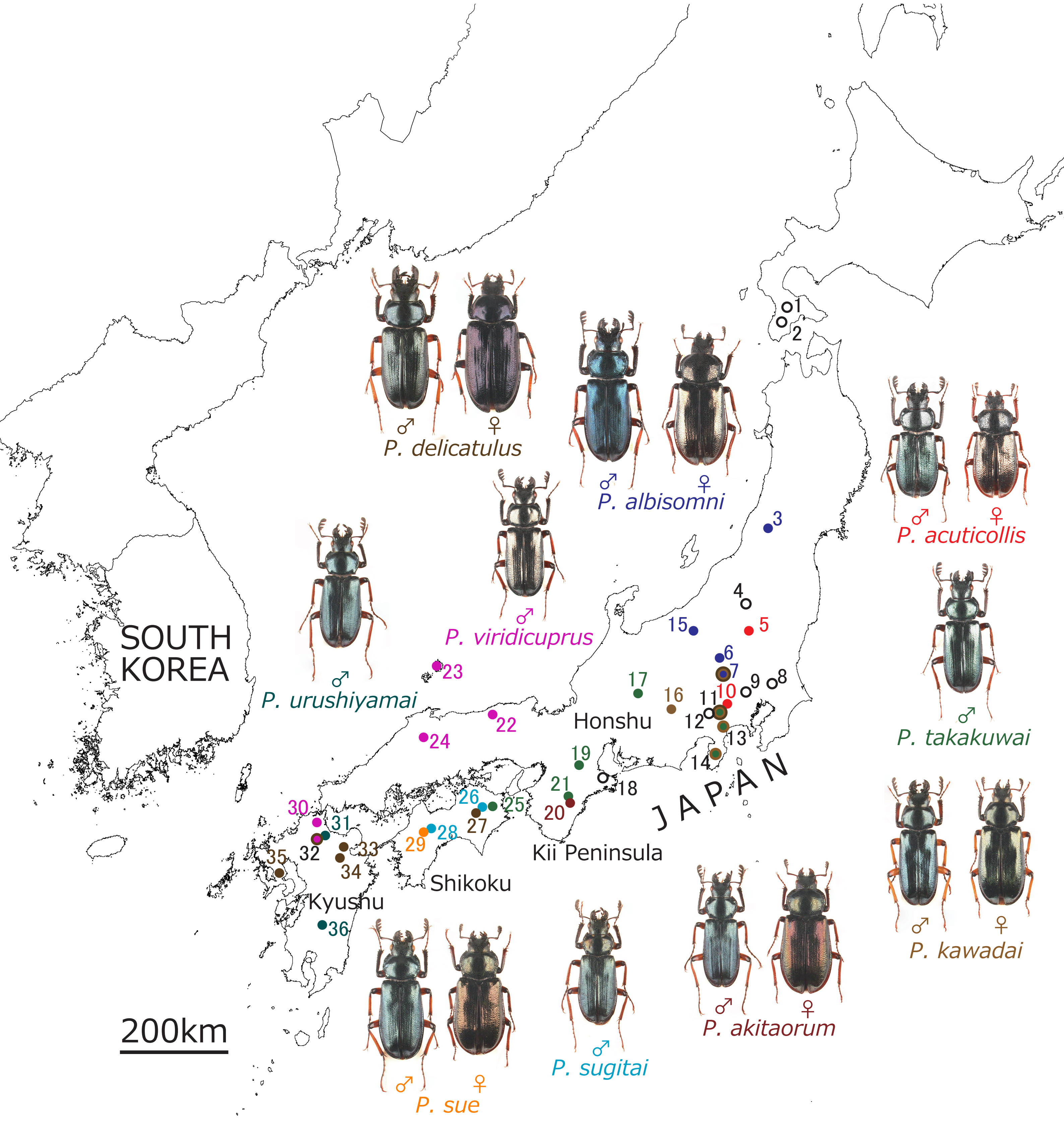

Supplementary Figure 1. Sample collection sites. Colored circles, collection sites of Japanese *Platycerus* species; open circles, collection sites of outgroup lucanid species (modified from Kubota et al. 2020). See Supplementary Tables 1, 2 for more information.
