# Supplementary material for: Diverse Heat Tolerance of the Yeast Symbionts of Platycerus Stag Beetles in Japan
Source: Front Microbiol. 2022 Jan 7;12:793592. doi: 10.3389/fmicb.2021.793592 (PMC8776712; doi:10.3389/fmicb.2021.793592)
Supplement: Supplementary file 5 [file Data_Sheet_5.PDF]

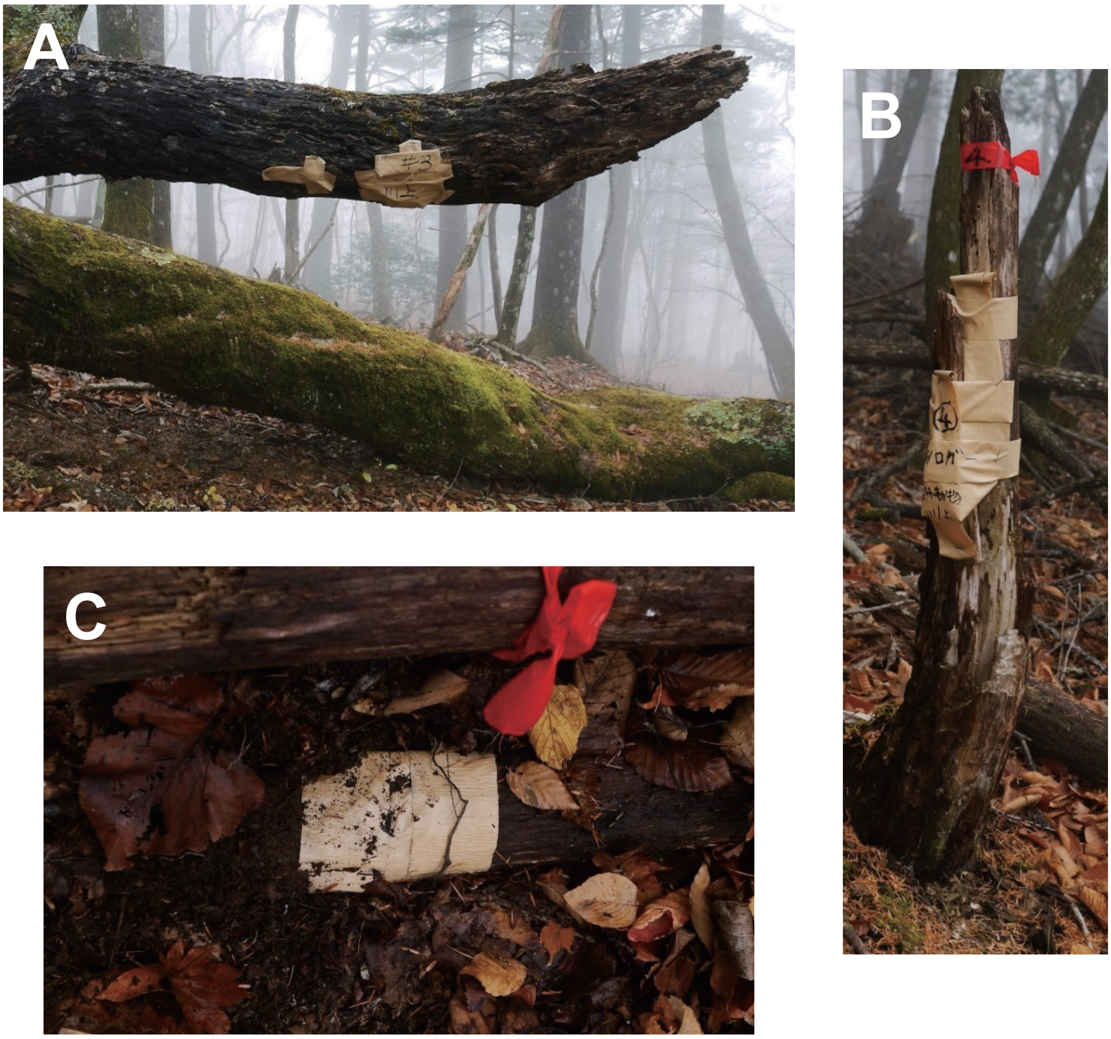

Supplementary Figure 2. Host wood materials of three sympatric Platycerus species in which temperature changes were investigated. (A), *P. delicatulus*; (B), *P. kawadai*; (C), *P. albisomni*.
