# Supplementary material for: Diverse Heat Tolerance of the Yeast Symbionts of Platycerus Stag Beetles in Japan
Source: Front Microbiol. 2022 Jan 7;12:793592. doi: 10.3389/fmicb.2021.793592 (PMC8776712; doi:10.3389/fmicb.2021.793592)
Supplement: Supplementary file 7 [file Data_Sheet_7.PDF]

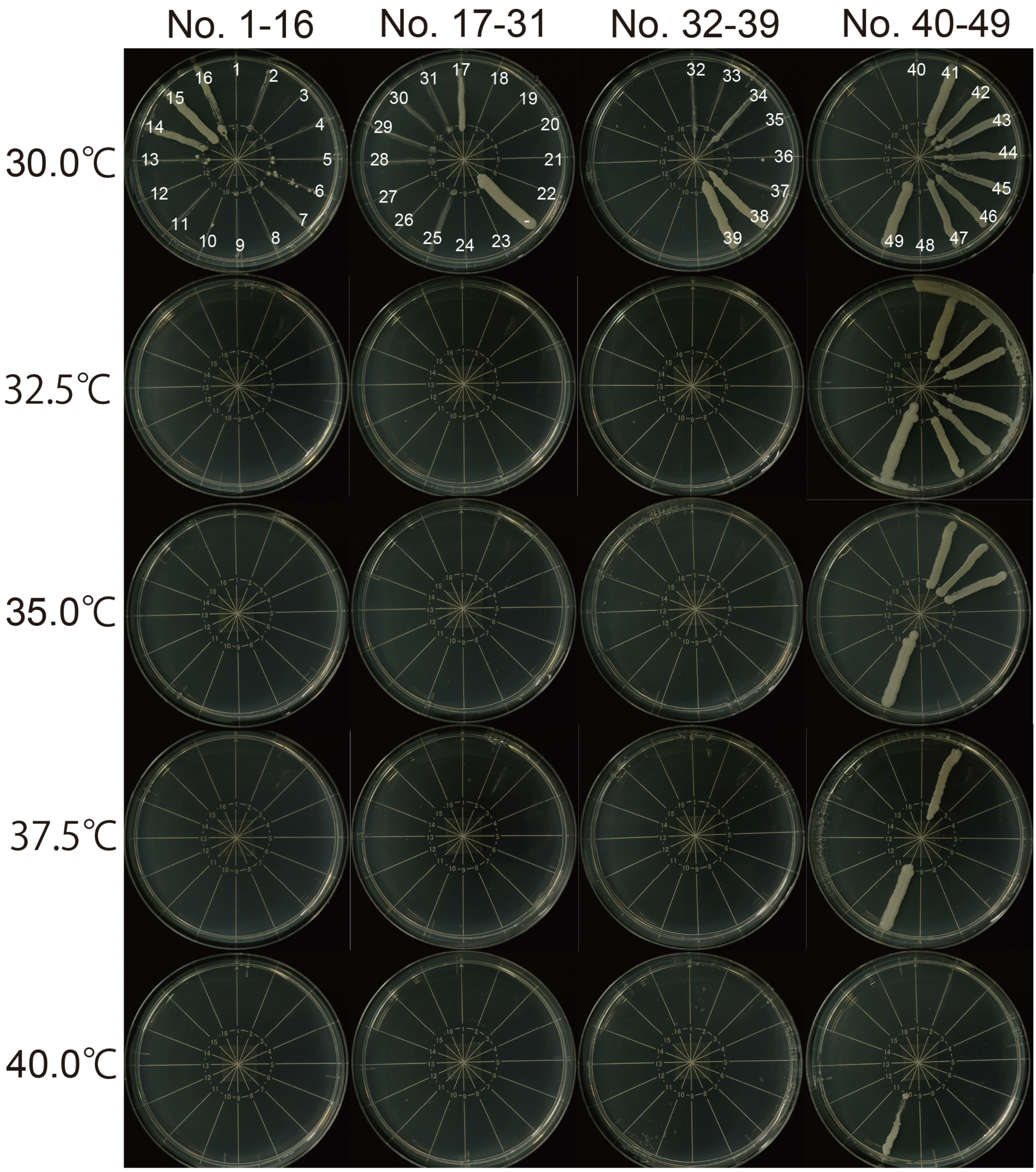

Supplementary Figure 4. Cultivating plate conditions at four days after the beginning of incubation at temperatures ranging from 30.0ºC to 40.0ºC, which were used to determine the maximum growth temperature (MGT) of *Scheffersomyces* yeasts. Numbers on the photographs indicate the strain number on each plate.
