# Supplementary material for: Diverse Heat Tolerance of the Yeast Symbionts of Platycerus Stag Beetles in Japan
Source: Front Microbiol. 2022 Jan 7;12:793592. doi: 10.3389/fmicb.2021.793592 (PMC8776712; doi:10.3389/fmicb.2021.793592)
Supplement: Supplementary file 9 [file Data_Sheet_9.PDF]

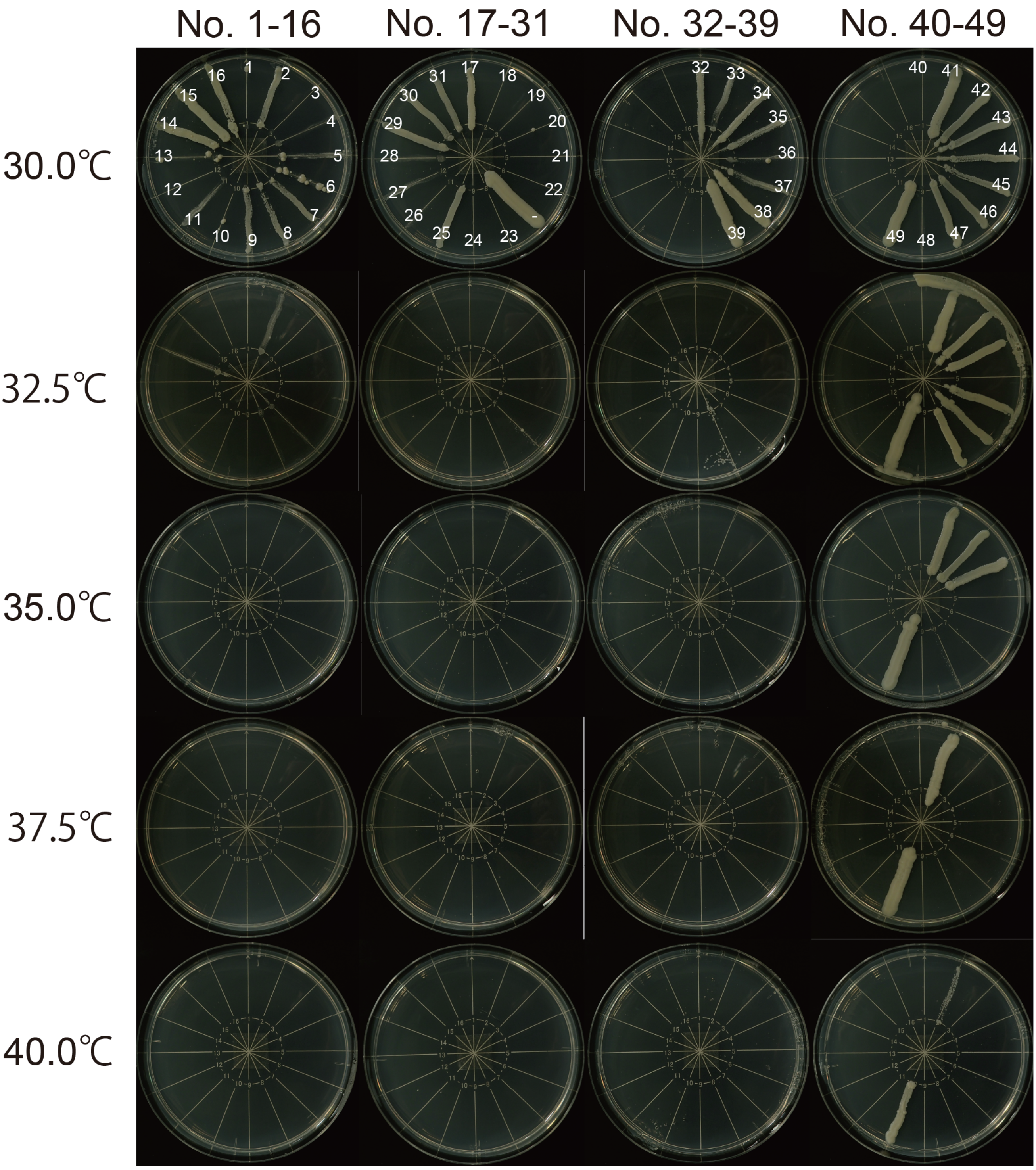

Supplementary Figure 6. Cultivating plate conditions at two days after the changing the incubation at temperature to 20.0ºC, which were used to determine the maximum survival temperature (MST) of *Scheffersomyces* yeasts. Temperatures on the left indicate the initial cultivating temperatures (30.0ºC–40.0ºC). Numbers on the photographs indicate the strain number on each plate.
