# Supplementary material for: Diverse Heat Tolerance of the Yeast Symbionts of Platycerus Stag Beetles in Japan
Source: Front Microbiol. 2022 Jan 7;12:793592. doi: 10.3389/fmicb.2021.793592 (PMC8776712; doi:10.3389/fmicb.2021.793592)
Supplement: Supplementary file 10 [file Data_Sheet_10.PDF]

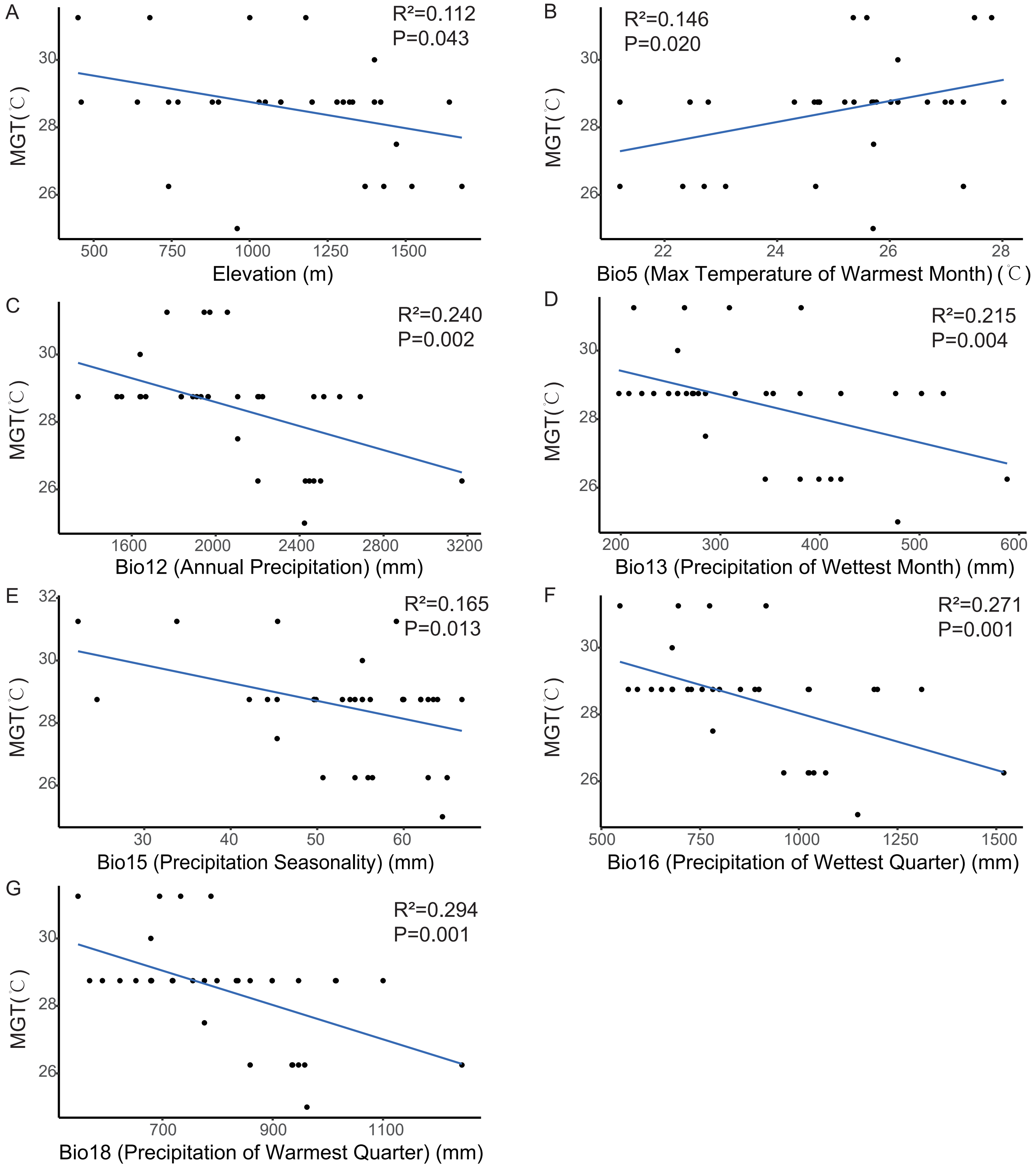

Supplementary Figure 7. Significant relationships between the maximum growth temperature (MGT) of *Platycerus* yeast symbionts and environmental variables.
