# Supplementary material for: Diverse Heat Tolerance of the Yeast Symbionts of Platycerus Stag Beetles in Japan
Source: Front Microbiol. 2022 Jan 7;12:793592. doi: 10.3389/fmicb.2021.793592 (PMC8776712; doi:10.3389/fmicb.2021.793592)
Supplement: Supplementary file 12 [file Data_Sheet_12.pdf]

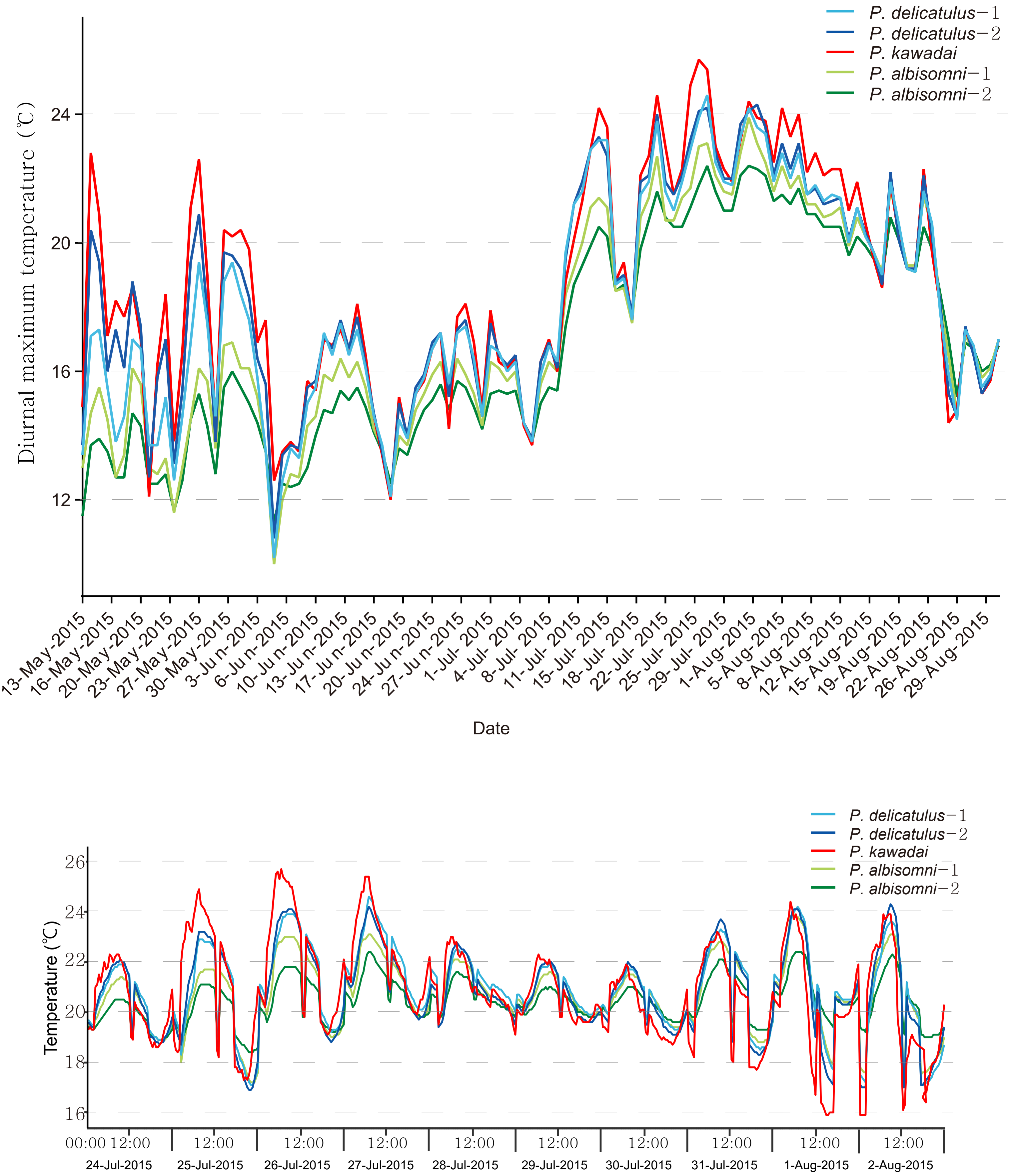

Supplementary Figure 9. Temperature change in host wood materials of three sympatric *Platycerus* species at Site 7, the Irikawa area, the University of Tokyo Chichibu Forest (1,300 m elevation). A, Diurnal maximum temperature change from May 13 to August 31, 2015; B, Temperature change from July 24 to August 2, 2015.
